# Neutralizing Monoclonal Antibodies to Fight HIV-1: On the Threshold of Success

**DOI:** 10.3389/fimmu.2016.00661

**Published:** 2017-01-11

**Authors:** Juan Pablo Jaworski, Alejandrina Vendrell, Sebastián Matias Chiavenna

**Affiliations:** ^1^National Scientific and Technical Research Council, Buenos Aires, Argentina; ^2^Institute of Virology, National Institute of Agricultural Technology, Castelar, Buenos Aires, Argentina; ^3^Pharmacological and Botanical Study Center, School of Medicine, University of Buenos Aires, Buenos Aires, Argentina; ^4^Ferrer Advanced Biotherapeutics, Barcelona, Spain

**Keywords:** human immunodeficiency virus, neutralizing antibodies, treatment, prophylaxis, reservoir, vaccine

## Abstract

Anti-human immunodeficiency virus type-1 (anti-HIV-1) neutralizing monoclonal antibodies are broadening the spectrum of pre- and post-exposure treatment against HIV-1. A better understanding of how these antibodies develop and interact with particular regions of the viral envelope protein is guiding a more rational structure-based immunogen design. The aim of this article is to review the most recent advances in the field, from the development of these particular antibodies during natural HIV-1 infection, to their role preventing infection, boosting endogenous immune responses and clearing both free viral particles and persistently infected cells.

## Neutralizing Antibody Response Against Human Immunodeficiency Virus Type-1 (HIV-1)

Human immunodeficiency virus type-1 displays the most effective evasion mechanisms described to date, including the following: (i) the expression of a reduced number of functional virus envelope proteins (Env) in the surface of the virion, (ii) a remarkable diversity, (iii) a dense sugar shield, and (iv) an extraordinary conformational flexibility, as it can be shown by the drastic conformational change of gp120 upon binding to the CD4 receptor. However, during HIV-1 natural infection, two different types of antibodies are produced by the host against Env. Binding antibodies (BAbs) arise within the first month after infection (1). These antibodies bind to non-functional Env present in the surface of the virion, and they are not able to block infection of target cells (2). Despite this limitation, several studies have shown that BAbs can modulate HIV-1 and SIV infection by killing infected cells through antibody-dependent cell cytotoxicity and antibody-dependent cell-mediated viral inhibition (3–7). On the other hand, neutralizing antibodies (NAbs) bind to functional Env and prevent the infection of target cells (8). At the beginning of the infection, NAbs are directed to immunodominant and mostly variable regions of Env, as it occurs with the third variable loop (V3) (9). Since they are only capable of neutralizing autologous viral variants, they are also called *strain-specific* or *autologous NAbs* (10).

During the course of the infection, continuous viral mutation and evasion constantly expose the immune system to novel, but related, HIV-1 antigens. This persistent antigenic stimulation provokes continuous selection of memory B-cells with higher affinity to Env. At a molecular level, the accumulation of somatic mutations provokes a closer conformational dependence between the NAbs and their specific epitopes. As a result of this, NAbs bind to more complex conformational structures, as is the case of the CD4-binding site (CD4bs) in gp120 (11). This particular contest between the immune system and HIV-1 results in an evolutionary race of unparalleled magnitude, which drives, in a subset of individuals, the production of NAbs capable of recognizing heterologous viral variants (10–15). At a clinical level, this provokes an increase in the NAb titers and the avidity of antibodies to Env (16).

## First Generation of Anti-HIV-1 Neutralizing Monoclonal Antibodies (NmAbs)

At the end of the 20th century, *in vitro* screening of plasma samples from HIV-1-infected individuals showed that a limited subset of plasmas neutralized a large number of heterologous HIV-1 variants. Despite technical limitations, some research groups were able to isolate anti-HIV-1 mAbs with broad neutralizing activity from these samples (Table 1) (17–21). Although not very potent, this first group of NmAbs (e.g., 447-52D, 4E10, 2F5, 2G12, b12) exposed some of the few weaknesses associated with HIV-1 Env. The antigenic determinants targeted by these NmAbs constituted conserved regions of Env. Since these structures were functionally involved (e.g., gp120 CD4bs, gp41 membrane-proximal external region, etc.) mutations at these points represented a high fitness cost to the virus (12, 22–26). Moreover, several research groups showed that passive transfer of these NmAbs blocked the infection with a chimeric simian immunodeficiency virus carrying HIV-1 Env (SHIV) in non-human primates (NHP) (27–34). These studies underscored the importance of the humoral component of the immune response as an effective prophylactic tool. Although numerous efforts were made in order to induce such type of response, the idea of an antibody-based vaccine did not succeed. One reason for that was that these first-generation NmAbs had some unusual characteristics as follows: (i) b12 was produced artificially from a phage library, (ii) 2G12 had atypical combinations of Fab segments, (iii) 2F5 and 4E10 were self-reactive, (iv) b12, 2G12, and 2F5 displayed modest breadth (<50%), and (v) 4E10 had low potency despite the fact of displaying broad neutralizing activity (<90%). Additionally, the amount of antibody necessary to protect macaques from infection was too high to be achieved through vaccination (18, 21, 35). Therefore, combination strategies and new, more potent antibodies were searched.

**Table 1:** **First and second generations of anti-HIV-1 neutralizing monoclonal antibodies (NmAbs)**.

Env subunit	Epitope region	NmAb name	Potency (*in vitro*)^a^	Breadth (*in vitro*)^b^	Protection efficacy (*in vivo*)^c^
Gp120	CD4-binding site (conformational epitope)	b12^d^ (18) VRC01 (01–03) (47) VRC07 (61) 3BNC117 (55, 60, 117) (44) NIH45-46 (44) G54W 12A12 (44) VRCPG04 (48, 142) VRC-CH31 (30–34) (48) HJ16 (54) N6	1.8 (47); 2.82 (45) 0.33 (45, 47); 0.22 (61); 0.3 (55); 0.9 (44) 0.11 (61); 0.16 (103) 0.134 (103); 0.11 (55) 0.2 (55); 0.41 (44) 0.04 (141) NC 0.196 (48); 0.2 (45) 0.098 (48) ND 0.038 (102)	17 (47); 10 (45, 46) 72 (47); 74 (45); 77 (61); 75 (55) 83 (61, 103) 79 (103); 77 (55) 76 (55) NC NC 64 (48); 65 (45) 70 (48) ND 96 (102)	25 mg/kg (4/4); 5 mg/kg (2/4) (28, 29, 34) 5 mg/kg (6/6); 0.3 mg/kg (5/12) (60) 0.2 mg/kg (3/4); 0.05 (0/4) (61) 5 mg/kg (3/4); 1 mg/kg (1/4) (62) 20 mg/kg (0/4) (62) ND ND ND ND ND ND
	
	V3 loop (lineal epitope)	447-52D^d^ (19) HGN194 (54)	NC NC	NC NC	ND ND
	
	V1/V2-glycan site (quaternary epitope linked to Asn160)	PG9 (46) PG16 (46) CH01-04 (143) PGT145 (141–145) (45)	0.23 (45, 46); 0.142 (103); 0.2 (55) 0.15 (46); 0.15 (55) NC 0.2 (45)	57 (46); 54 (45); 73 (103); 65 (55) 51 (46); 59 (55) 40 (143) 52 (45)	20 mg/kg (5/6); 5 mg/kg (3/6) (60) ND ND ND
	
	V3-glycan supersite (conformational epitope linked to Asn 332)	2G12^d^ (21, 144) PGT121 (121–123) (45, 120, 145) PGT128 (125–128) (45, 125) 10-1074 (45, 56) PGT130-131 (45) PGT133-134 (45) PGT135 (135–137) (45, 121)	2.38 (45, 46) 0.03 (45) 0.02 (45); 0.096 (103) 0.4 (103) 0.16–0.52 (45) ND 0.17 (45)	11 (45) 57 (45) 60 (45); 56 (103) 54 (103) ND ND 23 (45)	40 mg/kg (3/5) (27, 30, 33) 1 mg/kg (5/5): 0.2 mg/kg (3/5) (59) 10 mg/kg (5/5); 2 mg/kg (2/5) (146) 5 mg/kg (4/4) 1 mg/kg (1/4) (62) ND ND ND

Gp41	Gp41 MPER (lineal epitope)	4E10^d^ (17) 2F5^d^ (20) Z13 (26) 10E8 (55) HK20 (54)	3.41 (45, 46); 1.93 (55) 2.30 (46); 14.6 (55) ND 0.389 (103); 0.35 (55) ND	13 (45); 37 (55) 19 (46); 16 (55) ND 74 (103); 72 (55) ND	50 mg/kg (6/6) (31) 50 mg/kg (6/6) (27, 31, 33) ND 5 mg/kg (6/6); 0.3 mg/kg (3/6) (60) ND

Gp41-gp120 interfase	N-linked glycans adjacent to CD4bs and gp41 Quaternary structure of pre-fused gp41 Quaternary structure of pre-fused and cleaved gp41 Fusion peptide (gp41) and glycan at Asn88 (gp120) Quaternary structure of pre-fused gp41 and a glycan at Asn88 (gp120)	8ANC195 (44, 127) 35O22 (119) PGT151 (151–158) (116, 118) N123-VRC34.01 (57) 3BC176, 3BC315 (49, 147)	ND ND ND ND 1.69–10 (49, 147)	ND ND ND ND ND	ND ND ND ND ND

*^a^Median 50% inhibitory concentration (IC50) (μg/ml). Results were obtained from TZM-bl neutralization assay. Panels of 100–200 pseudoviruses, representative of all HIV-1 clades, were used to measure potency*.

*^b^Percentage of virus neutralized with an IC50 < 1 μg/ml. Results were obtained from TZM-bl neutralization assay. Panels of 100–200 pseudoviruses, representative of all HIV-1 clades, were used to measure breadth*.

*^c^Passive studies performed in non-human primates (NHP): indicated is the proportion of protected animals at each Ab dose*.

*^d^First-generation NmAbs*.

*ND, no data are available; NC, data available cannot be compared*.

## Second Generation of Anti-HIV-1 NmAbs

The fast development of new technologies made possible the isolation of new generation anti-HIV-1 NmAbs. First of all, high-throughput neutralization assays permitted the screening of a large number of plasma samples from HIV-1-infected patients. For this purpose, standardized panels of Env-pseudoviruses representing all major genetic subtypes of HIV-1 were included in the TZM-bl neutralization assay. The different viral strains were classified in Tier 1 (sensitive neutralization phenotype), Tier 2 (moderate), and Tier 3 (neutralization-resistant phenotype) based on their neutralization phenotype (36). Increased neutralization resistance observed in Tier 2, compared to Tier 1 viruses, was explained by the lower exposition of highly immunogenic epitopes in variable loops and co-receptor-binding domain of gp120. By using these single-cycle infection assay and standardized viral strain panels, breadth was defined by the percent of HIV-1 isolates that an antibody could neutralize at a fixed concentration. Additionally, potency was defined by the amount of antibody that inhibited 50 or 80% of a fixed virus inoculum (37). Starting in 2004, several groups began to report that 10–25% of HIV-1 patients could make antibodies that cross-neutralize many of the viral strain tested (11, 37, 38). A more recent study showed that 50% of sera from HIV-1 chronically infected subjects (*n* = 205) were able to neutralize more than 50% of viruses from a panel of 219 Env-pseudoviruses (39). However, only a small proportion (1%) of individuals were able to neutralize more than 80% of HIV-1 variants (11, 37, 40–48). These individuals are known as “elite neutralizers.”

Another technical advance consisted in the use of engineered protein probes such as the resurfaced stabilized core 3 to identify and sort epitope-specific memory B-cells from the plasmas of elite neutralizers (47). Alternatively, high-throughput microculture methods were used for B-cell direct neutralization screening (46). Either from single (43, 49–53) or cultured B-cells (45, 46, 54, 55), anti-Env mAbs were produced by highly sensitive genetic recovery of antibody heavy- and light-chain sequences. Altogether, these methodological advances allowed the discovery of a whole new generation of NmAbs, which displayed higher potency (~three orders of magnitude) and in most cases an expanded neutralization breadth (Table 1). These novel broad NmAbs (bNmAbs) shared most of Env specificities with the previous ones, as is the case of the CD4bs site (44, 47, 54), the glycan shield (45, 46, 56), and MPER (55). In addition, novel antigenic determinants were found such as the interphase between gp120 and gp41 trimeric structure, including one structure linked to the fusion peptide (57).

## New Generation bNmAbs Block HIV-1 and SHIV Infection

Similar to previous isolated bNmAbs, several studies showed that the passive transfer of this new generation bNmAbs blocked HIV-1 and SHIV infection both in humanized mice (hu-mice) and macaques, respectively (58–62). Moreover, a recent study revealed that a single 20 mg/kg dose of either VRC01-LS (63), 3BNC117, or 10-1074 prevented virus acquisition for up to 23 weeks, following weekly low-dose SHIV challenge regime (median = 14.5, 13, and 12.5 weeks, respectively) (64). Altogether, these studies highlight the effectiveness of these new bNmAbs as a potential pre-exposure prophylactic tool.

## New Generation bNmAbs Control HIV-1 and SHIV Chronic Infection

Although first-generation bNmAbs had shown only a modest to non-effect in the control of chronic HIV-1 infection, both in mice and humans (65–68), the discovery of second-generation bNmAbs prompted to re-test this approach. It was first demonstrated, by using a hu-mouse model, that a single dose of a triple combination of new generation bNmAbs (3BC176, PG16, and 45-46-G54W) suppressed HIV-1 viremia for 60 days after the cessation of treatment (69). Virus suppression was positively correlated with the antibody half-life, and the combination of bNmAbs avoided the appearance of resistant viral variants (69, 70).

When compared to hu-mouse, NHP models have overcome the impossibility of a direct challenge with HIV-1 viruses by using chimeric SHIVs that infect NHPs and can cause pathogenesis. The NHP/SHIV model allow to study infection in an intact immune system, and disease progression in this model resembles HIV-1 in several aspects: (i) high viral burden and diversity, (ii) presence of MHC-1 alleles involved in viral control, (iii) establishment of chronic infection, (iv) CD4+ T-cell loss, and (v) establishment of an immunodeficiency syndrome, etc. (71, 72). Using the NHP/SHIV model, two separate studies showed that a single administration of new generation bNmAbs to chronically infected macaques reduced viremia and cell-associated viral loads—in peripheral blood, gut mucosa, and lymph nodes—to undetectable levels for a period of 3–8 weeks (73, 74). In these studies, effective virus control was positively correlated with bNmAbs potency and half-life. Although in some cases monotherapy was associated with the appearance of viral escape mutants, the combination of bNmAbs with different specificities [i.e., PGT121 + 3BNC117 + b12 (73), or 3BNC117 + 10-1074 (74)] avoided this problem and increased treatment effectiveness. In addition, NmAb treatment in one of these studies improved the functionality of T-cell responses (73).

The positive outcomes observed in NHP and hu-mouse models prompted the testing of new generation bNmAbs in HIV-1 chronically infected patients. In this regard, two separate studies showed that the administration of a single dose of 3BNC117 or VRC01 reduced HIV-1 viremia (from 1 to 2.5 log10) for as long as 1 month, in patients who were not under combination antiretroviral treatment (cART) (75, 76). Additionally, two different studies have recently demonstrated that either 3BNC117 or VRC01 suppressed HIV-1 rebound following cART interruption in chronically infected patients (77, 78). However, the selection for preexisting and emerging resistant viral variants was reported in most of these studies, and consequently, single bNmAb therapy was not effective maintaining virus suppression in the long term (78). All in all, these studies highlight the benefits of new generation bNmAbs as a novel and efficient post-exposure treatment approach. Considering that virus suppression failed in patients with resistant viral variants, these results suggest that immunotherapy will require the combination of multiple bNmAbs that target different sites on HIV-1 Env for clinical use.

## New Generation bNmAbs Boost Endogenous Immune Response and Interfere with the Viral Reservoir

Combination antiretroviral therapy is effective in controlling HIV-1 viremia and preventing disease progression toward AIDS. However, lifelong treatment is required for the majority of patients. In addition to rapid virus dissemination and reservoir seeding, other immunological events that occur during HIV-1 acute infection affect the disease progression in the long term. This is the case of acute loss of CD4 memory T-cell located in the GUT-associated lymphoid tissue (79–83) and peripheral B-cell dysregulation (84–87). Although early cART can suppress viremia, reduce reservoir size, and restore immune function (88, 89), it fails to clear SIV infection even if started as early as 3 days post infection (90).

Antiretroviral drugs and bNmAbs limit HIV-1 infection by interfering with the viral life cycle. Additionally, bNmAbs can enhance host immune response by inducing the formation of immune complexes with the virus (91). Moreover, bNmAbs have the ability to promote the killing of HIV-1-infected cells through Fc-mediated cell cytotoxicity and phagocytosis (92). A recent study has shown that 3BNC117 enhanced host humoral immunity against HIV-1 (93). This result is in agreement with previous observations performed by Dr. N. Haigwood and colleagues (72, 94). Using a highly pathogenesis model in newborn macaques, Dr. Haigwood found that non-sterilizing levels of anti-SHIV-neutralizing IgG (SHIVIG) administered previous to oral challenge with SHIV_SF162P3_, reduced both plasma and peripheral blood mononuclear cell-associated viremia. Interestingly, SHIVIG also augmented and fastened the development of endogenous NAb response, which in turn correlated with lower set-point viremia and 100% survival of infected animals (72). Another recent study has revealed that passive administration of 3BNC117 accelerated the clearance of HIV-infected cells (95) by a mechanism that involved Fc gamma receptor (FcγR) engagement in a hu-mouse model. This observation is in correspondence with previous studies that had shown the importance of the Fc fraction of the antibody in the control of viremia, both in a hu-mouse (96) and an NHP model (28).

The benefits of antibodies compared to antiretroviral drugs prompted the testing of the therapeutic effect of the new generation of bNmAbs during acute infection. A first research group demonstrated that passive transfer of a cocktail of new generation bNmAbs (PGT121 and VRC07), administered prior to peak viremia (10 days post infection), suppressed SHIV viremia and limited the amount of cell-associated viral DNA in adult macaques (97). Moreover, NmAb therapeutic effect was similar to the one observed with cART initiated at the same time point (97). Although early NmAb treatment and cART were effective reducing the virus reservoir, none of these treatments removed it completely (97). In a second study performed by Hessell and colleagues, 1-month-old rhesus macaques were inoculated orally with SHIV_SF162P3_ (98). On days 1, 4, 7, and 10 after virus exposure, animals were injected subcutaneously (SC) with the same cocktail of bNmAbs (PGT121 and VRC07) used in the previous study. Replicating virus was found in multiple tissues by day 1 in animals with and without treatment. Remarkably, all NmAb-treated macaques were free of virus in blood and tissues at 6 months after exposure (98). Additionally, no anti-SHIV T-cell responses in blood or tissues at necropsy were detected and no virus emerged following CD8+ T cell depletion. Dr. Hessell’s observation concerning early viral clearance is in agreement with a latter study by Liu and colleagues, which showed that pre-exposure bNmAb-mediated protection against SHIV mucosal challenge might involve clearance of early viral foci in distal tissues (99). Altogether, these results suggest that early passive immunotherapy can eliminate early viral foci and thereby prevent the establishment of viral reservoirs.

## Current and Future Perspectives of HIV-1 Immunoprophylaxis

In the absence of an effective vaccine, passive administration of new generation anti-Env bNmAbs alone or in combination with cART has shown to be effective controlling and preventing HIV-1 infection. Moreover, latest research on the treatment of HIV-infected individuals has been heavily focused on developing strategies aimed to achieve sustained virologic remission without cART. In this regard, a series of phase I clinical trials have been designed to investigate the efficacy of new generation bNmAbs, such as VRC01. Although several bNmAbs have shown better performance compared with VRC01, the latter is considered the prototypic antibody from the new generation. In addition, the use of VRC01 in clinical trials is supported by substantial preliminary data showing the prophylactic and therapeutic efficacy of this antibody (47, 60, 61). The first large-scale clinical trial was performed in healthy adults in the United States. This trial showed that VRC01 was well tolerated by oral and SC routes. Furthermore, VRC01 had a terminal half-life of 15 days and pharmacokinetics comparable to other IgG (clearance 0.016 l/h) (100). After infusion, this antibody retained neutralizing activity and anti-VRC01 response was not detected following two mAb administrations. The results from a second clinical trial, performed in chronic infected patients who were undergoing treatment interruption, showed that VRC01 slightly suppressed plasma virus rebound, but it did not maintain virus control in the long term (78). Moreover, a rapid selection of preexisting and emerging resistant viral variants was reported in the same study. At this moment, VRC01 is in phase IIb efficacy trial as an intravenous infusion for HIV-1 prophylaxis. Future clinical trials planned for VRC01 include the following: (i) testing if VRC01 can control HIV-1 viremia in infected children and (ii) testing the efficacy of VRC01 to prevent mother-to-child transmission of HIV-1 (101). In addition to VRC01, novel VRC01-like antibodies with greater potency and breadth (>95%) have recently been isolated. By reducing the chance of selection for neutralization-resistant viral variants, novel antibodies such as N6 (102) might increase the efficacy of immunotherapy. Despite the promising features of the new isolated anti-Env bNmAbs, the extreme plasticity of HIV-1 demands permanent improvements in the field in order to guarantee complete success. In this regard, electron microscopy (EM), cryo-EM, and X-ray crystallographic studies have contributed to better understand the interaction between each NmAb and its epitope, prompting the rational design of more effective NmAb variants as is the case of VRC07 (VRC01 derived) and G54S (NIH45-46 derived) (Table 1). Bioengineered modifications of these antibodies have also increased their half-life and FcR function, augmenting their therapeutic window [e.g., VRC07 (61) and VRC01-LS (63)].

It has been demonstrated that co-administration of different bNmAbs that target distinct Env epitopes is important to achieve effective virus control without the emergence and/or selection of viral escape mutants. Different approximations based on *in vitro* neutralization data allowed to determine the best combination of bNmAbs (up to four) considering their breadth, potency, complete neutralization, and instantaneous inhibitory potential and countering escape variant production (103, 104). Alternatively, engineered bivalent anti-Env antibodies showed an exquisite HIV-1 neutralization activity while preserving normal architecture of IgG (105, 106). Ravetch and colleagues combined Fabs from two different bNmAbs, one to the CD4bs (3BNC117) and another to V3-glycan epitope (PGT135) using a special hinge domain that increases flexibility and favors intra-trimeric, heterobivalent crosslinking of the two Fab arms (107). This bispecific antibody performed far better than the combined activity of the individual parent antibodies [mean 50% inhibitory concentration (IC50) = 0.036 μg/ml and breadth = 93%]. This synergistic activity was equivalent to combined activity of up to five antibodies. A second engineered bispecific antibody, the 10E8V2.0/iMab showed an IC50 of 0.002 μg/ml and neutralized 99% of viral variants from a panel of 200 pseudoviruses (108). In this opportunity, the Fab from either anti-CD4 or anti-CCR5 mAb was linked to the Fab from the gp41-specific bNmAb 10E8. This construct increased 10E8 potency by anchoring the antibody on the CD4 T-cell membrane. Besides their remarkable *in vitro* performance, both bispecific antibodies reduced virus load substantially in HIV-1-infected humanized mice and also provided complete protection when administered prior to virus challenge (107, 108). A third construct was developed to link the Fab region of VRC07 bNmAb and an anti-CD3 mAb (109) in the same molecule. By engaging CD3, this molecule favored the expression of proviral genes in latently infected T-cells. At the same time, it mediated the killing of infected T-cells through the recognition of newly expressed Env. Overall, bispecific anti-HIV-1 Env antibodies showed improved breadth and potency and enhanced *in vivo* activity. These engineered antibodies represent an ideal therapeutic approach that would combine the breadth, the antigenic specificity, and the neutralization potency of two bNAbs into a single molecule, facilitating preclinical evaluation and development.

Alternatively to active and passive immunization, vector-mediated gene transfer could be used to secrete effective bNmAbs into circulation. This novel technique known as vectored immunoprophylaxis (VIP) is based on a specialized adeno-associated virus (AAV) vector optimized for the production of full-length antibody from muscle tissue. A couple of studies demonstrated that VIP was capable of protecting hu-mice from intravenous as well as vaginal challenge with diverse HIV strains (110, 111). Another study showed that VIP could maintain reduction of previously suppressed viral replication (70). Although this novel approach might overcome the limitations associated with passive transfer and active immunization (e.g., maintenance of the antibody concentration above protective level, immunogen design, sophisticated immunization regimens to induce extensive affinity maturation, etc.), there are some constraints associated with VIP that should be considered as follows: (i) preexisting immunity against the vector capsid might limit the efficiency of vector transduction, (ii) the route of administration might also affect the availability of antibody, (iii) the packaging limitation associated to AAV might influence the efficient delivery of both heavy and light chains of antibody, and (iv) there are safety uncertainties to be tested. Despite these limitations, VIP represents an interesting alternative for directly translating the existing repertoire of bNmAbs *in vivo*. Two different clinical trials are ongoing to test the safety and efficacy of VIP in humans (112). One of these trials, sponsored by the International Aids Vaccine Initiative is recruiting healthy males to receive AAV1 expressing PG9 bNmAb. A second trial carried out by the Vaccine Research Center (VRC) is testing AAV8 expressing VRC07. By either enhancing potency and breadth, or overcoming limitations of passive transfer of antibodies and vaccine design, these novel approaches might facilitate the use of bNmAbs into the clinic, for the prevention, control, and cure of HIV-1 infection.

## Lessons from bNmAbs Toward an Effective Vaccine Design

Thirty years after the discovery of HIV-1, the goal of developing a vaccine capable of eliciting a strong durable immune response to protect humans against the global diversity of HIV-1 isolates, remains elusive. However, in the last decade, important progress was made in this field with the moderate success achieved in a phase III clinical trial in Thailand (RV144 trial) leading the way. This approach consisted of four priming injections using a canarypox-vectored vaccine expressing HIV-1 Gag, Pol, and gp120 Env proteins (ALVAC-HIV), plus two boosts with soluble gp120 (AIDSVAX B/E) (113). This immunization regimen reduced the risk of acquiring infection in a 31.2% of individuals, and this was correlated with the presence of anti-V1 and -V2 antibodies (114). The vaccine also induced low level of neutralizing activity against Tier 1 viruses, meditated in large part by NAbs directed to V3 loop (115). In contrast, neutralization of Tier 2 viruses was not detected (115). Since Tier 2 viruses represent most circulating viral strains, the efficacy of RV144 might be improved by eliciting stronger NAb responses, particularly against Tier 2 viruses.

In addition to the partial success of the RV144 trial, other key findings underscored the importance of the humoral immune response against HIV-1. As described in previous sections, passively transferred bNmAbs have been shown to be effective blocking infection (58, 63, 64) and suppressing chronic SHIV and HIV-1 viremia (73–76). In addition, bNmAbs were able to potentiate endogenous antibody response (72, 93, 94) and mediate the clearance of SHIV and HIV-1 infection (95, 98, 99). These prophylactic and therapeutic properties of potent bNmAbs, together with the fact that they develop in 1% of infected individuals, support the rationale for developing an immunogen capable of inducing such type of response through vaccination.

In this regard, structural characterization has exposed how bNmAbs recognize Env, and the study of B-cell ontogenies are revealing pathways within the B-cell repertoire that lead to the eventual development of effective neutralizers (48, 116–132). Most bNmAbs targeting HIV-1 Env develop unusual features, including the frequent use of insertion and deletions and restricted germline use (133). They display extraordinary affinity maturation, reaching nucleotide somatic hypermutation (SHM) frequencies of 32 and 20% in heavy- and light-chain V genes, nearly double that of the normal SHM rates of other antibodies in the human repertoire (134) and can carry over 80 VH mutations (48, 54, 135). Another unusual feature of anti-Env bNmAbs is the presence of unusually long or short heavy-chain complementarity-determining region 3 (CDR3) loops. The PGT family of bNmAbs (Table 1) uses a long CDRH3 that makes it possible to trespass the dense glycan shield allowing interactions with gp120 peptides underneath. These particular features, necessary for the recognition of conserved conformational epitopes, are the result of long-term affinity maturation. This extraordinary maturation, which results from chronic stimulation of B-cells by mutating Env, provokes B-cell diversification from the germline toward Ag focusing (48).

Although these features are necessary to develop naïve B-cells into effective neutralizers, they might represent roadblocks to the development of bNmAbs during natural infection or vaccination. One example of this is that predicted germline precursors of VRC01- and PGT121-class antibodies lack detectable affinity for wild-type HIV-1 gp120, making it poor immunogen to induce a bNmAb response (136, 137). However, some research groups are devising strategies, using structure-based design of germline-targeting immunogens, to activate relatively rare VRC01-class precursors both in a transgenic mouse model expressing germline VRC01 heavy chain (138) and naïve B-cell from uninfected humans (139). Furthermore, boosting primed mice with specifically designed immunogens induced weak neutralization of fully native HIV-1 (140). Functional and structural analysis revealed that antibodies elicited were consistent with partially mature VRC01-like antibody. Following a similar rationale, Steichen and colleagues designed a stabilized Env trimer with affinity for germline-reverted precursors of PGT121-class bNmAbs to prime PGT121-like responses in PGT121 inferred-germline knockin mice (137).

Induction of effective bNmAbs will likely require a multi-step immunization strategy in which successive distinct boosting immunogens guide the genetic and functional maturation of bNmAbs. Whether or not the most effective strategies will follow a germline-targeting prime to drive antibody maturation toward a bNmAb phenotype is yet to be determined. Nonetheless, the continued pursuit of comprehensive studies of bNmAb structures and their interaction with HIV-Env and further investigations into the mechanisms involved in B-cell development and maturity that lead to the expansion of bNmAbs, constitute the basis for the rational design of novel immunogens to be included in an effective HIV-1 vaccine.

## Conclusion

New generation anti-HIV-1 bNmAbs are an important tool for the prevention, control, and eradication of HIV-1. A single pre-exposure dose of bNmAb is able to prevent SHIV infection for up to 6 months. When applied during chronic infection, a single dose of bNmAbs cocktail can control SHIV viremia and peripheral proviral loads to undetectable levels for up to 1 month, without the emergence of resistant variants. In addition, passively transferred bNmAbs are able to increase endogenous NAb response, modulating disease progression in the long term. Remarkably, if administered within the first 24 h of infection, bNmAbs cocktail can eliminate SHIV virus from the organism. The contribution of bNmAbs to killing HIV-1 persistently infected cells has also been demonstrated in a hu-mouse model, supporting the potential of bNmAbs to clear HIV-1 viral reservoir. It is an important fact that neither toxic nor anti-mAb responses have been reported in any of these studies. These results obtained in NHPs or hu-mice are currently being validated in human patients, and large-scale clinical trials have already been started to evaluate safety and efficacy of these antibodies. Furthermore, bNmAbs have directed the attention to functional structures of HIV-1 Env, exposing some weaknesses of this virus. The comprehensive analysis of the interaction between bNmAbs and these antigenic structures, in addition to the study of how B-cells develop to produce such type of antibodies, will contribute to the design of novel immunogens and immunization approaches.

## Author Contributions

JJ, AV, and SC performed the conception and design. JJ wrote the manuscript. JJ, AV, and SC revised and approved the manuscript.

## Conflict of Interest Statement

The authors declare no competing financial interest that could have influenced the content of this manuscript.
